# Implementierung eines sektorenübergreifenden Gesamtversorgungskonzeptes: Ergebnisse einer Sekundärdatenanalyse

**DOI:** 10.1007/s00391-021-01967-8

**Published:** 2021-08-30

**Authors:** Daniel Purwins, Martina Roes

**Affiliations:** 1 Deutsches Zentrum für Neurodegenerative Erkrankungen e.V. – Standort Witten, Stockumer Str. 12, 58453 Witten, Deutschland; 2 Department für Pflegewissenschaft, Fakultät für Gesundheit, Universität Witten/Herdecke, Witten, Deutschland

**Keywords:** Wandlungsmanagement, Stationäre Pflegeeinrichtung, Gesamtversorgungsvertrag, Wandlungsbereitschaft, Change management, Residential nursing home, Total care contract, Care delivery model, Willingness to change

## Abstract

**Hintergrund:**

Aufgrund einer gestiegenen Nachfrage nach ambulanten Leistungen von älteren Menschen aus der unmittelbaren Nähe zu seinen stationären Pflegeeinrichtungen erweiterte ein Träger sein (teil-)stationäres Pflegeangebot um ambulante Leistungen. Für die Realisierung einer solchen strategischen Neuausrichtung kommt es neben einer Orientierung an dem Wandlungsbedarf und der Wandlungsfähigkeit insbesondere auf die Wandlungsbereitschaft der Mitarbeiter des Trägers an.

**Ziel der Sekundärdatenanalyse:**

Identifikation von Faktoren, welche den Wandlungsbedarf, die Wandlungsfähigkeit und die Wandlungsbereitschaft der Mitarbeiter beeinflusst haben. Der Fokus dieses Artikels liegt auf der Wandlungsbereitschaft der Mitarbeiter.

**Material und Methoden:**

Basierend auf 32 leitfadengestützten (Einzel- bzw. Fokusgruppen‑)Interviews, die im Zeitraum von 2016 bis 2018 zu 3 verschiedenen Zeitpunkten mit 3 verschiedenen Mitarbeitergruppen des Trägers geführt wurden, erfolgte eine Sekundärdatenanalyse mittels inhaltlich-strukturierender qualitativer Inhaltsanalyse.

**Ergebnisse:**

Die Wandlungsbereitschaft der Mitarbeiter wurde durch folgende Faktoren beeinflusst: Die situationsunabhängige Veränderungsbereitschaft, die bewusste Entscheidung für das stationäre Setting, die Idee des Gesamtversorgungskonzeptes (GVK), die Umsetzung des GVK, die Leistungsempfänger, sowie Vorerfahrungen in der ambulanten Pflege.

**Diskussion:**

Die Ergebnisse bieten Einblicke dahingehend, welche Faktoren die Wandlungsbereitschaft von Mitarbeitern beeinflusst haben und, bezogen auf die Ausgestaltung vergleichbarer Veränderungsprozesse, zu berücksichtigen sind.

## Abstract (en)

**Background:**

Due to increased demand from older adults who live in the immediate vicinity of a few nursing homes, a large care provider in the community expanded its residential care service to include home care services. To be successful with such a strategy an orientation towards the needs and the ability to change as well as the willingness of employees to change is of exceptional importance.

**Aim of the secondary data analysis:**

Identification of factors that have influenced the need, the ability and eagerness for change. The focus of the article lies on the willingness of the employees to change.

**Material and methods:**

Based on 32 semi-structured interviews, which were conducted at 3 different times between 2016 and 2018 with 3 different groups of employees of the community care provider, a secondary data analysis was carried out using a content-structured qualitative content analysis.

**Results:**

Employees’ willingness to change was influenced by the following factors: the situational independent readiness for change (being ready for change regardless the situation?), conscious decision for the nursing home/ inpatient setting, the idea of the cross-sectoral care concept, the implementation of the cross-sectoral care concept, the recipients of nursing care/services, previous experience in home care.

**Conclusion:**

The results offer insights into which factors influence the willingness of employees to change. These factors influence change so that they need to be known and taken into account.

Um dem Wunsch älterer Menschen mit Hilfe- und oder Pflegebedarf nach einem möglichst langen Verbleib in der eigenen Häuslichkeit entsprechen zu können, haben quartiersbezogene Versorgungsansätze bedürfnis- und bedarfsgerechte Dienstleistungs- und Unterstützungsangebote zu berücksichtigen. Bezogen auf die Erbringung entsprechender Leistungen, kommt Einrichtungen der stationären Altenhilfe eine besondere Bedeutung zu, da sie bereits über einige für die Leistungserbringung notwendige Voraussetzungen (u. a. Mitarbeiter^1^ aus dem Bereich der Pflege, der Betreuung sowie der Hauswirtschaft und -technik) verfügen [1, 8, 11].

## Hintergrund

**Figure 1:**
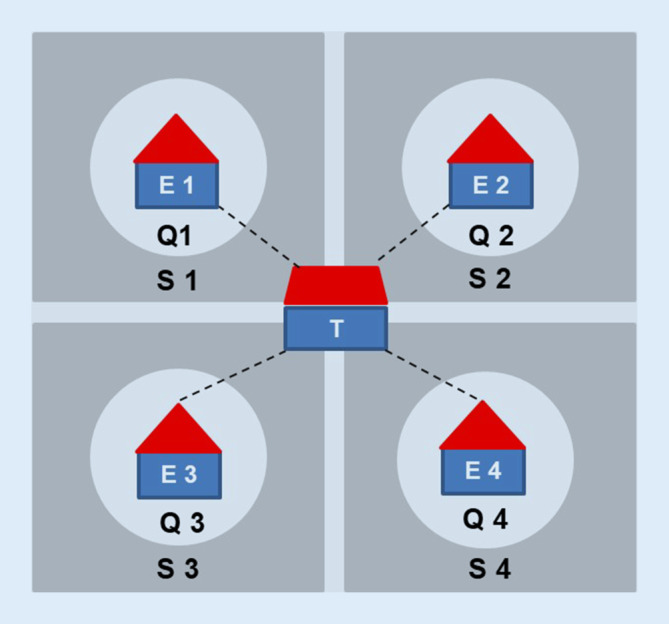

Ein Träger von 4 stationären Altenhilfeeinrichtungen in einer Stadt in NRW begann im Jahr 2014 damit, sein vorbestehendes (teil-)stationäres Leistungsangebot um quartiersnahe ambulante Leistungen zu erweitern. Die Erweiterung des Leistungsangebots erfolgte vor dem Hintergrund von zunehmend häufigeren Anfragen älterer Menschen aus dem näheren Umfeld der stationären Pflegeeinrichtungen nach ambulanten pflegerischen Versorgungsangeboten (SGB V und SGB XI) sowie niedrigschwelligen Unterstützungsleistungen (u. a. betreuungsbezogene Leistungen, Einkäufe, Gartenarbeit) [4]. Die Leistungserbringung im ambulanten Bereich erstreckt sich auf einen regional anhand von Straßenzügen definierten Bereich in der unmittelbaren Umgebung zu den Einrichtungen der stationären Altenhilfe (Quartier) und nicht auf die gesamten Stadtteile, in denen sich die jeweiligen Einrichtungen des Trägers befinden (Abb. 1; [4]).

Die Leistungserbringung im ambulanten Bereich erfolgt durch Mitarbeiter aus den stationären Einrichtungen heraus, sodass auf die Gründung eines klassischen ambulanten Pflegedienstes verzichtet wurde. Dieses Charakteristikum verdeutlicht, dass es, bezogen auf die Umsetzung des Ansatzes, insbesondere auf die Bereitschaft der von der Veränderung betroffenen Mitarbeiter des Trägers ankam, auch Leistungen im ambulanten Bereich zu erbringen. Der Leistungsausweitung lag die Idee zugrunde, vielfältige Leistungen aus einer Hand zu erbringen. Diese Idee wurde in einem „Gesamtversorgungskonzept“ (GVK) verschriftlicht. Für die Umsetzung des GVK schloss der Träger einen Vertrag zur Erbringung von häuslicher Krankenpflege gemäß § 132 SGB V sowie für jede stationäre Einrichtung einen Gesamtversorgungsvertrag gemäß § 75 Abs. 1 SGB XI ab [4, 15].

Die mit der Umsetzung des GVK einhergehende Leistungsausweitung kann als eine strategische Neuausrichtung des Träger der stationären Altenhilfeeinrichtungen betrachtet werden. Krüger [9] zufolge hat die Umsetzung einer solchen strategischen Neuausrichtung an 3 Koordinaten orientiert zu erfolgen: dem Wandlungsbedarf, der Wandlungsbereitschaft und der Wandlungsfähigkeit.

## Fragestellung, Forschungsdesign und methodisches Vorgehen

### Primärstudie.

Die Umsetzung und Bewertung des GVK war im Zeitraum von 2015 bis 2018 Gegenstand einer von der Stiftung Wohlfahrtspflege NRW finanziell geförderten Primärstudie mit der Bezeichnung „Vielfalt aus einer Hand“. Die Primärstudie wurde als eine multiperspektivische Evaluationsstudie konzipiert. Die 4 verschiedenen Perspektiven wiesen unterschiedliche Schwerpunkte, Zielsetzungen sowie (quantitative und qualitative) methodische Vorgehensweisen auf [4]. Bei den Perspektiven handelte es sich um (a) die Perspektive der hilfe- oder pflegedürftigen Menschen im ambulanten Bereich und ihrer versorgenden Angehörigen (Nutzerperspektive), (b) die Perspektive der Mitarbeiter des Trägers der Pflegeeinrichtungen mit und ohne Führungs‑/Leitungsfunktion (Dienstleisterperspektive), (c) die Perspektive der Akteure des regionalen Versorgungssystems (Systemperspektive) sowie (d) die aus der Umsetzung des GVK resultierenden ökonomischen Konsequenzen (ökonomische Perspektive) [4].

Für die in diesem Beitrag dargestellte Sekundärdatenanalyse wurde auf Daten zurückgegriffen, die in der „Dienstleisterperspektive“ der Primärstudie erhoben wurden. Die übergeordnete Fragestellung der Dienstleisterperspektive lautete: „Wie gestaltet sich die konkrete Umsetzung des Konzepts in der pflegerischen Versorgung?“ [4, S. 13]. Zur Beantwortung dieser Fragestellung wurde im Zeitraum von 2016 bis 2018 in jeder der 4 Pflegeeinrichtungen zu 3 Zeitpunkten leitfadengestützte Interviews (entweder Einzel- oder Fokusgruppeninterviews) mit 3 (T0 & T2) bzw. 2 (T1) verschiedenen Mitarbeitergruppen geführt (Tab. 1). Die nach einer informierten Einwilligung geführten Interviews wurden digital aufgezeichnet, transkribiert und für den Zweck der Primärstudie in Form einer strukturierenden qualitativen Inhaltsanalyse [4] analysiert.

**Table 1:** 

Erhebungen	Personengruppen	Anzahl der Teilnehmer pro Interview und Pflegeeinrichtung			
		E1	E2	E3	E4
T0	Leitungen	1	2	1	1
	Amb. Leistungserbringer	3	2	3	3
	Stat. Leistungserbringer	3	3	3	3
T1	Leitungen	–	–	–	–
	Amb. Leistungserbringer	5	6	3	4
	Stat. Leistungserbringer	2	3	2	3
T2	Leitungen	2	2	4	2
	Amb. Leistungserbringer	3	3	4	4
	Stat. Leistungserbringer	2	3	3	4

*E1–4:* Einrichtungen (durchnummeriert von 1 bis 4)

*Leitungen:* Die Leitung(en), die in den Einrichtungen am stärksten in die Umsetzung des Gesamtversorgungskonzeptes involviert ist (sind) (ggf. Einrichtungsleitung und oder verantwortliche Pflegefachkraft)

*Amb. Leistungserbringer:* Mitarbeiter, die neben der Arbeit im stationären Bereich auch Leistungen im ambulanten Bereich erbringen

*Stat. Leistungserbringer:* Mitarbeiter, die auf den Wohnbereichen arbeiten, aus denen heraus ambulante Leistungen erbracht werden. Die Mitarbeiter selbst erbringen jedoch keine ambulanten Leistungen

### Sekundärdatenanalyse.

Das Ziel der Sekundärdatenanalyse bestand darin, den Prozess der strategischen Neuausrichtung des Anbieters von Pflegeleistungen zu analysieren. Hierzu wurde auf die theoretisch begründeten Koordinaten des Wandlungsmanagements [9] zurückgegriffen: den Wandlungsbedarf (d. h. das aus unternehmensexternen und -internen Faktoren resultierende sachliche Ausmaß notwendiger Veränderungen), die Wandlungsbereitschaft (d. h. die innere Einstellung gegenüber dem Wandel sowie die Bereitschaft, aktiv am Wandel mitzuwirken) sowie die Wandlungsfähigkeit (d. h. die auf personen- und systembezogenen Faktoren basierende Möglichkeit, Wandlungsprozesse zu realisieren) [9, 13]. Basierend auf diesen Koordinaten wurden folgende Forschungsfragen formuliert: Woraus resultiert der Wandlungsbedarf? Welche Faktoren beeinflussen die Wandlungsbereitschaft der Mitarbeiter? Welche Merkmale kennzeichnen die Wandlungsfähigkeit? In diesem Artikel liegt der Fokus auf der Wandlungsbereitschaft der Mitarbeiter, da dieser eine besondere Bedeutung in Veränderungsprozessen zukommt [2, 3, 9, 10].

Basierend auf den Transkripten der in der Dienstleisterperspektive geführten leitfadengestützten Einzel- bzw. Fokusgruppeninterviews (s. oben) wurde eine Sekundärdatenanalyse mittels inhaltlich-strukturierender qualitativer Inhaltsanalyse [16] durchgeführt. Das Kategoriensystem wurde in einer gemischt deduktiv-induktiven Vorgehensweise erarbeitet, indem für die Oberkategorien, ausgehend von den Fragestellungen, auf die 3 Koordinaten des Wandlungsmanagements [9] zurückgegriffen wurde und die Unterkategorien induktiv aus dem Material heraus generiert wurden. Das von dem Erstautor kontinuierlich am Material und basierend auf Gesprächen mit der Zweitautorin modifizierte und weiterentwickelte Kategoriensystem [21] wurde in einem Kodierhandbuch verschriftlicht. Die mit der Entwicklung des Kategoriensystems korrespondierende Kodierung des Materials erfolgte ebenfalls durch den Erstautor. War eine Zuordnung von Kodiereinheiten zu den Kategorien des Kategoriensystems nicht eindeutig möglich, so wurden diese mit der Zweitautorin kritisch reflektiert und konsentiert. Zudem wurde die Kodierung des gesamten Materials 2‑mal vollständig durch den Erstautor überprüft und bei Bedarf angepasst.

## Ergebnisse

### Stichprobenbeschreibung

Insgesamt wurden 3 leitfadengestützte Einzelinterviews und 29 leitfadengestützte Fokusgruppeninterviews geführt (Tab. 1).

### Wandlungsbereitschaft der Mitarbeiter: beeinflussende Faktoren

**Figure 2:**
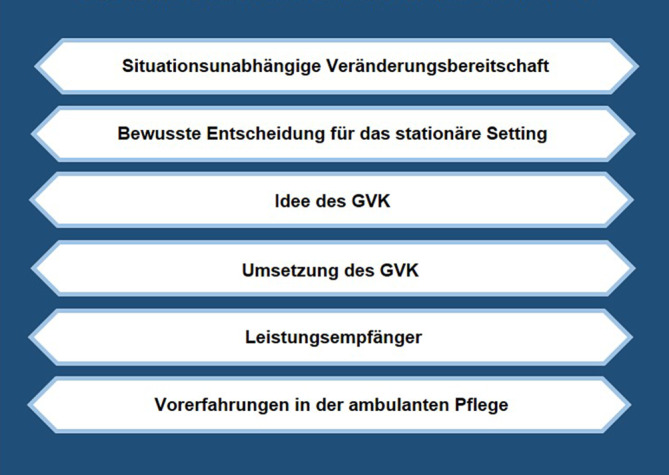

Im Folgenden werden die Ergebnisse, bezogen auf die Determinanten der Wandlungsbereitschaft, dargestellt (Abb. 2).

#### Situationsunabhängige Veränderungsbereitschaft*.*

Diese resultiert aus persönlichen Eigenschaften wie beispielsweise Neugierde, grundsätzlicher Aufgeschlossenheit gegenüber Veränderungen oder auch Abneigungen gegenüber Routinen: „*Ich bin auch so ein Mensch, ich brauche immer wieder mal Veränderungen. Und so Routine ist für mich katastrophal. Und deswegen finde ich das auch toll, dass es gerade so eine starke Veränderung gibt*“ (Q2, T1, A, K03.01, 457–459). Auch wurde auf das Gegenteil verwiesen, d. h. auf eine grundsätzlich negative Einstellung gegenüber Veränderungen, die u. a. durch Schwierigkeiten, sich auf Veränderungen einlassen zu können, oder auch Ängste/Befürchtungen vor Veränderungen gekennzeichnet sei: „[…] *Veränderungen machen Angst, ich bin auch nicht gerade der Veränderungstyp, ich hasse Veränderungen. Aber ist nun mal so*“ (Q4, T1, A, K03.01, 1107–1108).

#### Bewusste Entscheidung für das stationäre Setting.

Die bewusste Entscheidung für die Arbeit in einer Einrichtung der stationären Altenhilfeeinrichtung und damit gegen die Arbeit bei einem ambulanten Pflegedienst resultiert den Interviewpartnern zufolge aus unterschiedlichen Gründen: dem Wunsch, in einer stationären Einrichtung zu arbeiten, der persönlichen Stärke im Bereich der stationären Altenhilfe, Vorerfahrungen in der ambulanten Pflege oder auch den Arbeitsanforderungen der ambulanten Pflege: *„Ich war nie ambulant und wollte auch nie ambulant, deswegen hab’ ich mich bewusst irgendwann mal für einen stationären Bereich entschlossen“ (Q4, T1, A, K03.02, 1077–1078)*.

#### Idee des GVK.

Aus Sicht der *Leistungserbringer* scheint die dem GVK zugrunde liegende Idee für gut befunden zu werden, da das GVK bisher einzigartig sei, die Innovativität des Unternehmens zum Ausdruck bringe und dazu führe, dass sich das Unternehmen positiv von der Konkurrenz unterscheide: *„Das ist doch was Tolles, was wir da anbieten … Das können die anderen Häuser nicht machen … Doch da heben wir uns ab, das ist ja vom Sinn her super“ (Q3, T0, S, K03.03, 1011–1016)*. Zudem trage das GVK zu der Schaffung neuer Arbeitsplätze und erweiterter Aufgabenbereiche für die Mitarbeiter bei.

Im Hinblick auf die *Leistungsempfänger* scheint die dem GVK zugrunde liegende Idee von den Interviewpartnern positiv bewertet zu werden, da Menschen trotz Hilfe‑/Pflegebedürftigkeit ermöglicht werden könne, in ihrer eigenen Häuslichkeit zu verbleiben: *„A: Was ich halt an diesem Konzept auch gut finde, ist einfach, dass wir Menschen in so einem kleinen Quartier die Möglichkeit bieten, halt einfach auch zu Hause zu bleiben“ (Q2, T1, A, K03.03, 1061–1063).* Auch hätten ambulante Klienten im Zeitverlauf (bei zunehmender Hilfs‑/Pflegebedürftigkeit) die Möglichkeit, in eine stationäre Pflegeeinrichtung des Unternehmens zu ziehen.

Gegen die Idee des GVK spreche den Interviewpartnern zufolge u. a., dass das GVK keine neuen Ideen für den stationären Bereich beinhalte, sowie das Empfinden, dass die Versorgung der ambulanten Klienten wichtiger sei als die Pflege der Bewohner in der stationären Einrichtung: *„Nur, was mir so am Herzen liegt, ist, … ich möchte nicht, dass die ambulante Pflege, so hört sich das nämlich manchmal an, wichtiger wird wie unser stationärer Bereich […] Ja, aber es hört sich ganz oft … so an: ‚Das ist wichtig, […] das müsst ihr machen, das gehört jetzt dazu‘. Ich höre nicht mehr, ‚passt aber auf, dass es stationär alles läuft‘. […] Das ist das, was mich eigentlich stört“ (Q2, T2, A, K03.03, 580–588)*.

#### Umsetzung des GVK.

Die Umsetzung des GVK lässt sich durch mehrere Aspekte näher beschreiben, die Auswirkungen auf die Wandlungsbereitschaft hatten: (a) Die Art und Weise des Vorgehens der Führungs‑/Leitungskräfte, bezogen auf die Umsetzung des GVK. (b) Die gewählte Organisationsform zur Vereinbarung der ambulanten und der stationären Leistungserbringung: *„Ich habe auch eine sehr kritische Einstellung dazu, weil ich tatsächlich denke, dass die Gesamtstruktur noch nicht passt, deswegen wollte ich von vornherein nicht darin arbeiten […]“ (Q3, T2, S, K03.04b, 56–58)*. (c) Die aus der Organisationsform resultierenden veränderten Arbeitsanforderungen der Mitarbeiter: *„Es ist wieder ein ganz anderes Arbeiten, ne. Also ich finde, körperlich hast du es nicht so schwer wie jetzt stationär“ (Q2, T2, A, K04.04d, 171–172)*. (d) Das soziale System in Form des Kollegenkreises, inkl. ihrer Reaktionen auf die vorgenommenen Veränderungen, und/oder die aus diesen resultierenden Konsequenzen/Ergebnisse: *„[Interviewpartner A:] Mir macht das nichts aus, dass ich rausgehe, aber ich freue mich auf jeden Tag, wenn ich nicht raus muss und auf der Station bleiben kann. [Interviewpartner B:] Siehst du, und ich mach das noch nicht und höre das. Dann werde ich natürlich nicht hingehen und sagen, ‚das will ich auch‘“ (Q2, T1, S, K04.04c, 270–273).* (e) Die für die Mitarbeiter sichtbaren Ergebnisse des Wandels, die beispielsweise verdeutlichen, dass die Veränderungen zu den erhofften Ergebnissen geführt haben: *„Aber letzten Endes, dass die [ambulanten Klienten] dann irgendwann mal auch unsere Kunden werden, auch hier stationär. Das hat sich ja tatsächlich bewährt. Also […] das ist schon ’ne gute Sache“ (Q4, T1, A, K03.04g, 1156–1158)*; oder auch Ergebnisse, die von Interviewpartnern nicht als erstrebenswert erachtet werden, weil sie sich ihrem Empfinden nach negativ auf die Möglichkeit auswirken, Leistungen im stationären Bereich erbringen zu können oder zu einer erhöhten Arbeitsbelastung führen. (f) Die Möglichkeit, einen bestehenden Teilzeitarbeitsvertrag aufstocken zu können, oder auch die Notwendigkeit, für die Leistungserbringung im ambulanten Bereich einen zusätzlichen Arbeitsvertrag (auf 450 € Basis) abschließen zu müssen. (g) Die mit dem Arbeitsvertrag korrespondierende Möglichkeit, beim eigenen Arbeitgeber zusätzlich Geld hinzuverdienen zu können.

#### Leistungsempfänger.

Positiv auf die Wandlungsbereitschaft wirken sich den Interviewpartnern zufolge die von den Mitarbeitern als „nett“ wahrgenommenen ambulanten Klienten, ihre Dankbarkeit für die erbrachten Leistungen sowie die Wahrnehmung aus, dass Angehörige von ambulanten Klienten durch die Leistungserbringung entlastet werden können: *„Und …, ja, ich find’s einfach positiv, dass man sieht, dass man eigentlich […] mit ’ner morgendlichen Grundpflege […] ’ne Entlastung halt bringen kann“ (Q3, T0, L, K04.05, 809–811)*. Eher negativ auf die Wandlungsbereitschaft wirken sich den Interviewpartnern zufolge hingegen folgende Phänomene aus: (a) die Anforderung an die Mitarbeiter, das häusliche Umfeld der ambulanten Klienten so zu akzeptieren, wie es ist, (b) das Gefühl der Mitarbeiter, (b1) dass in der stationären Altenhilfeeinrichtung durch die Organisation zur Vereinbarung ambulanter und stationärer Leistungserbringung (und einer damit korrespondierenden, häufig als zu gering empfundenen personellen Besetzung im stationären Bereich) weniger Zeit für die Versorgung der Bewohner zur Verfügung stehe, (b2) dass die Versorgung der ambulanten Klienten als wichtiger empfunden werde als die der Bewohner in der stationären Altenhilfeeinrichtung, und (b3) nicht zu wissen, wie es den ambulanten Klienten gehe, wenn diese nach Abschluss der Leistungserbringung alleine in ihrer Häuslichkeit verbleiben: *„Im Ambulanten, wenn ich die Türe zu mache, dann ist das schon mal ein komisches Gefühl, die allein zu lassen. Ne, wo man dann denkt: ‚auweia, hoffentlich geht das heute gut‘, oder dass man sich darüber Gedanken macht, und das ist für mich selber unangenehm“ (Q4, T1, A, K04.05, 524–527)*.

#### Vorerfahrungen in der ambulanten Pflege.

Bezogen auf die Vorerfahrungen verdeutlichen die Aussagen der Interviewpartner zweierlei: Zum einen, dass sich positive Vorerfahrungen in der ambulanten Pflege positiv auf die Bereitschaft auswirken, Leistungen im ambulanten Bereich zu erbringen: *„Das Ambulante ist sowieso […] so meins, und das liegt mir auch, und das macht mir enorm Spaß […]“ (Q2, T0, A, K04.06, 789–791)*. Zum anderen, dass Vorerfahrungen in der ambulanten Pflege dazu geführt haben, dass sich Mitarbeiter bewusst für die Arbeit in einer stationären Altenhilfeeinrichtung entschieden haben: *„Ich hatte auch meine Praktika, zweimal sechs Wochen [in der ambulanten Pflege], und da hab’ ich gesagt, ‚nee, also das möchtest du nicht machen. Du möchtest auf jeden Fall stationär arbeiten‘“ (Q3, T0, A, K03.06, 927–928)*.

## Diskussion

Die in diesem Beitrag dargestellten Ergebnisse der Sekundärstudie verdeutlichen, dass insgesamt 6 Themen von besonderer Relevanz bezogen auf die Wandlungsbereitschaft der Mitarbeiter sind: (1) Die insbesondere aus persönlichen Eigenschaften resultierende situationsunabhängige Veränderungsbereitschaft, die von Krüger [9] und Freyth [3] bereits als ein die Wandlungsbereitschaft beeinflussender Faktor beschrieben wurde. (2) Die bewusste Entscheidung der Mitarbeiter für das stationäre Setting, die sich u. a. vor dem Hintergrund nachvollziehen lässt, dass sich die Anforderungen im ambulanten Bereich von denen im stationären Bereich deutlich unterscheiden und Pflegende sich häufig aufgrund ihrer Präferenzen explizit für die Arbeit in einer stationären Altenhilfeeinrichtung entscheiden [17]. Indem die Arbeit in den 2 Settings der pflegerischen Versorgung (ambulant und stationär) jeweils mit unterschiedlichen Anforderungen an die Mitarbeiter verbunden ist, geht die Umsetzung eines settingübergreifenden Versorgungsansatzes folglich mit Implikationen auf die Auswahl, die Qualifizierung und den Einsatz der Mitarbeiter einher. Bezogen auf die Pflegenden bleibt abzuwarten, inwieweit die generalistische Pflegeausbildung [12] eine ausreichende Antwort auf die skizzierten Anforderungen darstellt. (3) Die Idee des GVK, was als Bestätigung betrachtet werden kann, dass der konkrete Inhalt/Gegenstand des Wandlungsvorhabens die Wandlungsbereitschaft beeinflusst [3]. (4) Das Vorgehen bei der Umsetzung des GVK, was als Bestätigung der u. a. von Kauffeld und Ebner [6, S. 405] beschriebenen Auffassung verstanden werden kann, dass die Art und Weise, wie Veränderungsprozesse gestaltet werden, „[…] die Einstellung der Betroffenen gegenüber den geplanten Veränderungen und deren Unterstützungsaktivitäten“ beeinflusst. (5) Die Leistungsempfänger, bezogen auf die u. a. deutlich wurde, dass durch die Organisation zur Vereinbarung ambulanter und stationärer Leistungserbringung bei den Mitarbeitern z. T. das Gefühl entstand, weniger Zeit für die Versorgung der stationären Bewohner zur Verfügung zu haben, und sich dieses Gefühl negativ auf die Wandlungsbereitschaft der Mitarbeiter auswirkt. Dies lässt sich vor dem Hintergrund nachvollziehen, dass eine zentrale Aufgabe professionell Pflegender in der Verantwortungsübernahme („advocacy“) für die pflegebedürftigen Personen besteht [5] und diese Verantwortungsübernahme durch weniger Zeitressourcen als bedroht wahrgenommen werden kann. (6) Vorerfahrungen, die in der ambulanten Pflege gesammelt wurden und die sich auf die Bereitschaft der Mitarbeiter auswirken. Dies kann beispielsweise als Bestätigung betrachtet werden, dass sich insbesondere positive Erfahrungen, bezogen auf den Gegenstand des Wandels, auch positiv auf die Wandlungsbereitschaft auswirken [3].

Eine nähere Betrachtung der Ergebnisse verdeutlicht, dass – abgesehen von der situationsunabhängigen Veränderungsbereitschaft – alle Faktoren (die bewusste Entscheidung für das stationäre Setting, die Idee des GVK, die Umsetzung des GVK, die Leistungsempfänger und die Vorerfahrungen in der ambulanten Pflege) in einem Zusammenhang mit der konkreten Situation, d. h. dem GVK und seiner Umsetzung, stehen und folglich übergeordnet als situationsabhängige Faktoren bezeichnet werden können. Damit weisen die dargestellten Ergebnisse auf übergeordneter Ebene ein Merkmal auf, das in der Literatur bereits beschrieben wurde. So unterscheiden Krüger [9], bezogen auf die Wandlungsbereitschaft, zwischen der situationsunabhängigen und der situationsabhängigen Wandlungsbereitschaft und Freyth [3], bezogen auf die Veränderungsbereitschaft, zwischen einer allgemeinen und einer spezifischen Veränderungsbereitschaft.

Vor dem Hintergrund, dass sich die dargestellten Ergebnisse ausschließlich auf die Mitarbeiter des eingangs beschriebenen Trägers von Pflegeeinrichtungen beziehen und es aufgrund der Komplexität von Unternehmen nicht möglich ist, „Patentrezepte“ für die Gestaltung von Wandlungsprozessen zu formulieren [7, S. 5], lassen sich die Ergebnisse nicht ohne Weiteres auf die strategischen Neuausrichtungen anderer Träger übertragen. Es ist jedoch möglich, mit den dargestellten Ergebnissen auf Aspekte hinzuweisen, die mit der Wandlungsbereitschaft der Mitarbeiter korrespondieren. Diese Aspekte sollten Träger von Pflegeeinrichtungen kennen, die ebenfalls an einer Ausweitung ihres vorbestehenden stationären Leistungsangebots um quartiersnahe ambulante Leistungen interessiert sind, um sie bei ihrer Planungs- und Umsetzungskonzeption berücksichtigen zu können.

Die Altenhilfe wird auch in Zukunft weiteren Veränderungen unterliegen, die von Leistungserbringern auszugestalten sein werden und das berufliche Selbstverständnis der Mitarbeiter betreffen werden [18]. So existieren bereits verschiedene Reformvorschläge [14, 19, 20, 22], die z. T. deutlich über die Ambulantisierung stationärer Einrichtungen und sektorenübergreifender Gesamtversorgungsverträge hinausgehen und beispielsweise eine Pflegepolitik auf kommunaler Ebene vorsehen [19, 20].

## Fazit für die Praxis

Die Ausweitung von (teil-)stationären Pflegeleistungen um quartiersnahe ambulante Leistungen kann auf unterschiedlichen Wegen erfolgen. Eine Möglichkeit der Leistungsausweitung stellt das in diesem Beitrag dargestellte Gesamtversorgungskonzept (GVK) dar. Die Umsetzung eines GVK stellt einen tiefgreifenden Wandlungsprozess dar, der orientiert am Wandlungsbedarf, der Wandlungsbereitschaft und der Wandlungsfähigkeit realisiert werden sollte.

Die Bereitschaft der am Wandel beteiligten und vom Wandel betroffenen Mitarbeiter ist, bezogen auf die Umsetzung eins GVK, von zentraler Bedeutung. Die in diesem Beitrag dargestellten Ergebnisse verdeutlichen, dass die Wandlungsbereitschaft durch mehrere Faktoren beeinflusst wurde: die situationsunabhängige Veränderungsbereitschaft, die bewusste Entscheidung von Mitarbeitern für das stationäre Setting, die Idee des GVK, die Umsetzung des GVK, die Leistungsempfänger und Vorerfahrungen von Mitarbeitern in der ambulanten Pflege. Diese Faktoren gilt es von den für die Gestaltung von Veränderungsprozessen verantwortlichen Akteuren zu kennen und zu berücksichtigen, um die Bereitschaft der vom Wandel betroffenen und am Wandel beteiligten Mitarbeiter nicht negativ zu beeinflussen.

## Footnotes

1 In dem vorliegenden Beitrag wird bei geschlechtsspezifischen Begriffen in der Regel die maskuline Schreibweise gewählt. Dies soll lediglich der besseren Lesbarkeit dienen und nicht als Diskriminierung verstanden werden.
